# Determination of antihistaminic drugs alcaftadine and olopatadine hydrochloride via ion-pairing with eosin Y as a spectrofluorimetric and spectrophotometric probe: application to dosage forms

**DOI:** 10.1186/s13065-024-01137-y

**Published:** 2024-02-22

**Authors:** Sayed M. Derayea, Khalid M. Badr El-din, Ahmed S. Ahmed, Ahmed A. Khorshed, Mohamed Oraby

**Affiliations:** 1 Department of Pharmaceutical Analytical Chemistry, Faculty of Pharmacy, Minia University, Minia, 61519 Egypt; 2 Department of Pharmaceutical Analytical Chemistry, Faculty of Pharmacy, Sohag University, Sohag, 82524 Egypt; 3 Department of Biomedical Engineering, University of Alberta, Edmonton, AB T6G 1H9 Canada

**Keywords:** Alcaftadine, Olopatadine hydrochloride, Eosin Y, Spectrophotometry, Spectrofluorimetry, Pharmaceutical formulation

## Abstract

Four sensitive and fast analytical approaches relied on ion pairing with eosin Y were built up and evaluated using spectroscopy for determination of Alcaftadine and Olopatadine hydrochloride with high sensitivity and selectivity. Two spectrofluorimetric techniques were employed to observe the quenching effect of Alcaftadine or Olopatadine hydrochloride on the intrinsic fluorescence of eosin Y in a 0.1 M acetate buffer solution at pH 3.8 and 3.3 for Alcaftadine and Olopatadine hydrochloride, respectively. Those methods are considered the first spectrofluorimetric methods for Alcaftadine and Olopatadine hydrochloride assay. The fluorescence quenching effect was linear with concentration ranging from 150 to 2000 and 200 to 2000 ng mL^−1^ for Alcaftadine and Olopatadine hydrochloride, respectively. In the two spectrophotometric techniques, the absorbance of the produced ion-pair was monitored at 548 and 547 nm in aqueous buffered solution at pH 3.8 and 3.3 for Alcaftadine and Olopatadine hydrochloride, respectively. Beer's law was obeyed in the concentrations range of 0.8–8.0 and 1.0–10.0 µg mL^−1^. The four techniques were evaluated in accordance with ICH requirements and were effectively used to analyze dosage forms with a high percent recovery.

**Supplementary Information:**

The online version contains supplementary material available at 10.1186/s13065-024-01137-y.

## Introduction

**Fig. 1:**
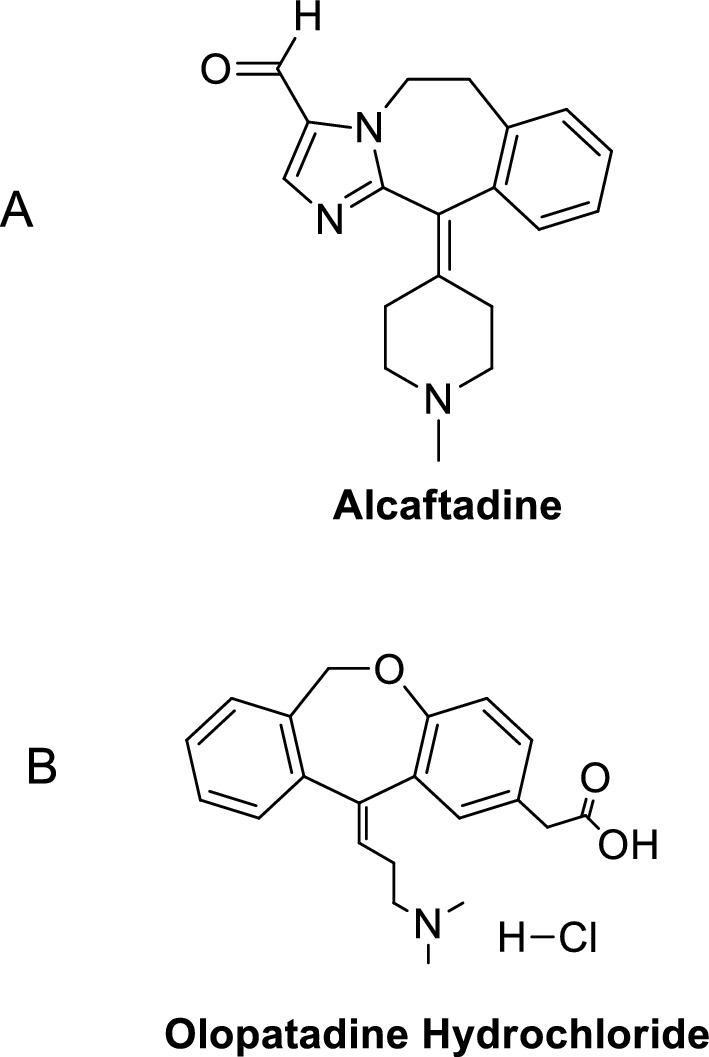
**A** chemical structure of ALC and **B** chemical structure of OLO

Alcaftadine (ALC) is an antihistaminic and mast cell stabilizer medication used to treat allergic conjunctivitis itching. It also has modulatory function on immune cell mobilization and the stabilizing effects of mast cells. It acts by preventing histamine release from mast cells [1]. Olopatadine hydrochloride (OLO) is an antihistaminic that acts as a specific H1 receptor antagonist. These medicines specifically bind to H1 receptors, preventing indigenous Histamine from acting. It is used to relieve irritation from allergic conjunctivitis. It's also used to treat allergic rhinitis, eczema dermatitis, chronic urticaria, cutaneous pruritis, psoriasis vulgaris and multiform erythema exsudativum [2, 3]. The chemical formula for ALC is 6, 11-dihydro-11-(1-methyl-4-piperidinylidene)-5H-imidazo [2, 1-b] [3] benzazepine-3-carboxaldehyde (Fig. 1A) [1]. The chemical formula for OLO is 11-[(Z)-3- (dimethylamino) propylidene]-6,11-dihydrodibenz [b,e] oxepin- 2-acetic acid monohydrochloride (Fig. 1B) [4]. Many methods were reported for assay of ALC and OLO using a variety of techniques, including spectrophotometry [5–10], HPLC [7, 11–14], LC–MS [4, 15], and HPTLC [7, 16–19]. These methods either lack sensitivity for spectrophotometric methods or lack simplicity for chromatographic methods. In spite of its high sensitivity and simplicity, no spectrofluorimetric technique was reported for determining ALC or OLO until now.

Dyes derived from xanthene, like erythrosine B and eosin Y, have been employed in chemical analysis of various drugs [20–22]. Eosin Y, classified as an acidic dye, represents the disodium form of 2,4,5,7-tetrabromo-fluoresceine. The dye was utilized for the selective estimation of some drugs in pharmaceutical preparations and biological fluids [22]. In addition, ion pairing with eosin Y provide a green spectroscopic approach for drug analysis [20, 21] Therefore, eosin Y had been extensively utilized to quantify numerous basic lipophilic medicines by forming ion pair complexes using spectrophotometric or spectrofluorimetric methods [23].

Compared to other previously published techniques for ALC or OLO, the spectrofluorimetric approaches have the benefits of being simple, sensitive, and not require sophisticated instrument or tedious preparation of samples. It also does not use excessive harmful organic solvents [24–26]. On the other hand, since the absorbance was measured in the visible region, colorimetry is preferable than UV-spectrophotometry because it avoids potential interfering effects of pharmaceutical excipients and common organic solvents [27].

Therefore, the current paper describes the first spectrofluorimetric methods in addition to spectrophotometric methods for rapid and easy determining ALC and OLO with high sensitivity and selectivity. The four techniques are free from extraction with harmful solvents and were constructed in accordance with green chemistry. Two green assessment tools were employed to evaluate the environmental friendliness of the current procedures: the Green Analytical Procedure Index (GAPI) [28] and the Analytical Greenness Calculator (AGREE) [29]. The suggested spectrofluorimetric methods are based on the reduction of eosin Y native fluorescence when ALC or OLO is added, while the increase in the absorbance was monitored in the case of the spectrophotometric methods.

## Experimental

### Instrumentation

The spectrofluorimetric measurements were performed with JASCO FP-8350 spectrofluorometer. The instrument has a 150 W Xe-arc lamp and a PMT adjusted to a voltage of 400 V. The slit width was 5 nm for the emission and excitation monochromators, and the scanning rate was 1000 nm per min.

The spectrophotometric measurements were performed with a T80 double beam UV–VIS spectrophotometer (PG instruments, Leicestershire, UK) coupled to UV-Win software. The measurements were made in quartz cells with a diameter of one centimeter.

Double-distilled water was made using Aquatron water still a4000d (Cole-Parmer, Staffordshire, UK).

### Materials and reagents

ALC was kindly supplied by Orchidia Pharmaceuticals (Al-Obour, Cairo, Egypt). The purity of ALC was found to be 99.60% ± 0.30 according to the reported method [8]. OLO was kindly supplied by EIPICO (Tenth of Ramadan City, Egypt). The purity of OLO was found to be 99.25% ± 0.12 as estimated using the official method [14]. Orchinohist® eye drops labeled to contain 0.25% of ALC (B.N. 10-1219137), obtained from Orchidia Pharmaceuticals (Al-Obour, Cairo, Egypt). Olohistine® eye drops labeled to contain 0.1% of OLO (B.N. 2006321), obtained from EIPICO (Tenth of Ramadan City, Egypt). Conjyclear forte® eye drops labeled to contain 0.2% of OLO (B.N. 10-0721176) and Conjyclear forte® single dose units (SDU) eye drops labeled to contain 0.2% of OLO (B.N. 10-0721146) obtained from Orchidia Pharmaceuticals (Al-Obour, Cairo, Egypt). Eosin Y was obtained from Merck (Darmstadt, Germany). Solutions of 2.5 × 10^–5^ and 1.04 × 10^–3^ M from Eosin Y were prepared in distilled water for the spectrofluorometric and spectrophotometric measurements for ALC, respectively. Solutions of 5.0 × 10^–4^ and 1.0 × 10^–3^ M of Eosin Y were prepared for the spectrofluorometric and spectrophotometric measurements for OLO, respectively.

Spectroscopic grades methanol, ethanol, and acetonitrile were supplied from Merck (Darmstadt, Germany). Analytical grade dimethyl formamide (DMF) was supplied from Fischer Scientific (Loughborough, U.K). Analytical grade ethyl acetate, acetone, acetic acid and sodium acetate were supplied by El Nasr pharmaceutical Chemical Co. (Cairo, Egypt). Solutions of 0.1 M sodium acetate and 0.1 M acetic acid were made up to prepare the acetate buffer solution.

### Preparation of standard solution

Fifty milligrams of ALC and OLO were dissolved in 500 mL double distilled water in separate flasks to make stock standard solutions (100 μg mL^−1^) for the spectrophotometric determination. Further dilution with distilled water was performed to prepare stock standard solutions (20 μg mL^−1^) for the spectrofluorometric determination.

### Procedures for general assay

#### Spectrofluorimetric method for ALC (Method A)

Standard solutions of ALC in concentrations ranging from 1.5 to 20 µg mL^−1^ were poured to volumetric flasks with 10 mL capacity, then 1.0 mL of 0.1 M acetate buffer (pH 3.8) was added. After that, 1.2 mL of 2.5 × 10^–5^ M eosin Y reagent was added. Finally, the flasks were totaled to the mark using distilled water and the contents were mixed thoroughly. The intensities of the fluorescence of the resulting solutions were monitored at 540 nm after exciting at 302 nm. A blank experiment was processed using the previous procedure except adding the drug solution. The quenching values of the fluorescence intensity of eosin Y solution were plotted against the concentrations of ALC to construct the calibration plot.

#### Spectrophotometric method for ALC (Method B)

Standard solutions of ALC in concentrations ranging from (8–80 µg mL^−1^) were moved into volumetric flasks with 10 mL capacity, then 1.0 mL of 0.1 M acetate buffer (pH 3.8) was added, followed by 1.7 mL of 1 × 10^–3^ M eosin Y reagent. Finally, the flasks were completed to 10 mL using distilled water. The content was shacked thoroughly. The absorbance of the resulting solutions was scanned at 548 nm. A blank experiment was processed using the previous procedure except adding the drug solution.

#### Spectrofluorimetric method for OLO (Method C)

Standard solutions of OLO in concentrations ranging from 1 to 20 µg mL^−1^ were poured to volumetric flasks with 10 mL capacity, then 0.5 mL of 0.1 M acetate buffer (pH 3.3) was added. After that, 1.3 mL of 5 × 10^–4^ M eosin Y reagent was added. Finally, the flasks were totaled to the mark using distilled water and the contents were mixed thoroughly. The intensities of the fluorescence of the resulting solutions were monitored at 546 nm after exciting at 303 nm. A blank experiment was processed using the previous procedure except adding the drug solution. The quenching values of the fluorescence intensity of eosin Y solution were plotted against the concentrations of OLO to construct the calibration plot.

#### Spectrophotometric method for OLO (Method D)

Standard solutions of OLO in concentrations ranging from (10–100 µg mL^−1^) were moved into volumetric flasks with 10 mL capacity, then 0.3 mL of 0.1 M acetate buffer (pH 3.3) was added, followed by 1.2 mL of 1 × 10^–3^ M eosin Y reagent. Finally, the flasks were completed to 10 mL using distilled water. The content was shacked thoroughly. The absorbance of the resulting solutions was scanned at 547 nm. A blank experiment was processed using the previous procedure except adding the drug solution.

### Estimation of the stoichiometric ratio between ALC and eosin Y

The reaction stoichiometry between ALC and eosin Y was investigated using Job's method [30]. Equi-molar solutions (1.3 × 10^–3^ M) of both ALC and eosin Y were prepared. Series of 1.0 mL portions of mixtures of 0.1:0.9, 0.2:0.8, 0.3:0.7, 0.4:0.6, 0.5:0.5, 0.6:0.4, 0.7:0.3, 0.8:0.2 and 0.9:0.1 of ALC (1.3 × 10^–3^ M): eosin Y (1.3 × 10^–3^ M) were mixed in 10 calibrated flasks and 1.0 mL of 0.1 M acetate buffer solution (pH 3.8) was added. The volume of the flasks was completed to the mark with double distilled water. The absorbance was measured at 548 nm and the obtained absorbance was corrected relative to the reagent blank readings for each eosin Y concentration. Job`s plot was constructed by plotting the corrected absorbance against the mole fraction of ALC.

### Estimation of the stoichiometric ratio between OLO and eosin Y

Equi-molar solutions (1.3 × 10^–3^ M) of both OLO and eosin Y were prepared of both OLO and eosin Y. Solutions composing of complementary volumes of both reactants with total volumes of 1.0 mL were mixed in 10 calibrated flask and 0.3 mL of 0.1 M acetate buffer solution (pH 3.3) was added. The volume of the flasks was completed to the mark with double distilled water. The absorbance was measured at 547 nm and the obtained absorbance was corrected relative to the reagent blank readings for each eosin Y concentration. Job’s plot was constructed by plotting the corrected absorbance against the mole fraction of OLO.

### Procedure for the estimation of ALC and OLO in pharmaceutical formulation

Aliquot of 0.2 mL of eye drops was diluted in a 10-mL calibrated flask using double distilled water as the primary dilution solution. For the spectrofluorimetric method, 0.2 mL of resulted solution was further manipulated in a 10-mL calibrated flask to get working solutions. While, for the spectrophotometric method, 1.0 mL of resulted solution was further manipulated in a 10-mL calibrated flask.

An aliquot of 0.4 mL of Olohistine® eye drops or 0.2 mL of Conjyclear forte® eye drops or Conjyclear forte® SDU eye drops was diluted in a 10-mL calibrated flask using double distilled water as the primary dilution solution. For the spectrofluorometric method, 0.3 mL of resulted solution was further diluted in a 10-mL calibrated flask to obtain the working solutions. While, for the spectrophotometric method, 0.2 mL of resulted solution was further diluted in a 10-mL calibrated flask.

## Results and discussion

**Fig. 2:**
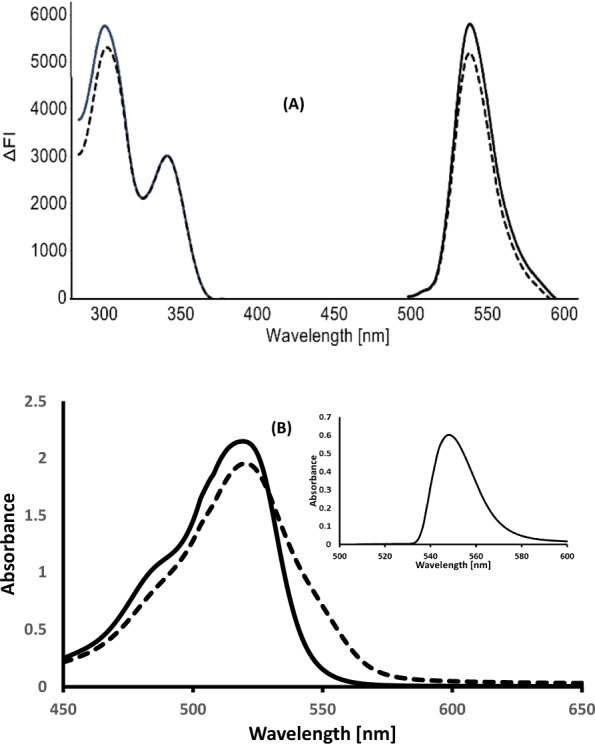
**A** Excitation and emission spectra of eosin 2.5 × 10^−5^ M (▬) and its reaction with 1500 ng mL^−1^ALC (-----) **B** Absorbance spectra of the blank (▬) and its reaction with 6 µg mL^−1^ ALC **- - - -**, and the insert show the absorbance spectrum of the complex after subtracting the eosin blank

**Fig. 3:**
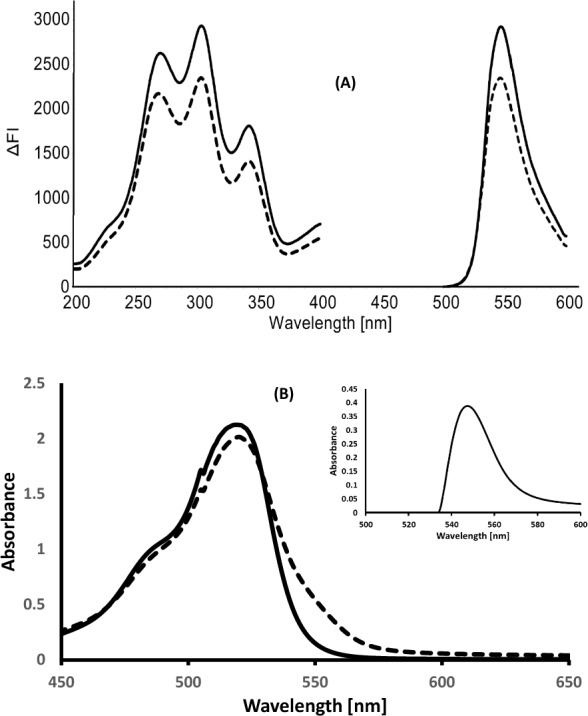
**A** Emission spectra of eosin 5 × 10^–4^ M (▬) and its reaction with 1200 ng mL^−1^ OLO (------). **B** Absorbance spectrum of the blank (▬) and its reaction with 8 µg mL^−1^ OLO reaction (**- - - - -**), and the insert show the absorbance spectrum of the complex after subtracting the eosin blank

Eosin Y is an acidic dye that is commonly utilized as a probe for the fluorometric and spectrophotometric analysis of some amino compounds [23]. The reaction between the amino groups of ALC with anionic form of eosin Y at the suitable pH (3.8 in the spectrofluorometric and spectrophotometric methods) using acetate buffer led to the formation of the association complex. The reaction between the amino groups of OLO with the anionic form of Eosin Y at pH 3.3 for the spectrofluorometric and the spectrophotometric methods, using acetate buffer led to the formation of the association complex. The suggested spectrofluorimetric method (A) is based on measuring the reduction of Eosin Y native fluorescence at 540 nm after excitation at 302 nm upon adding ALC (Fig. 2A), while an increase in absorbance at 548 nm in the case of the spectrophotometric method (B) (Fig. 2B). The suggested spectrofluorometric method (C) is based on the reduction of Eosin Y native fluorescence upon the addition of OLO at 546 nm after excitation at 303 nm upon adding OLO (Fig. 3A). While in the spectrophotometric method (D), the increase in absorbance was monitored at 548 nm (Fig. 3B).

### Optimization of the experimental conditions

#### Effect of buffer volume and pH

**Fig. 4:**
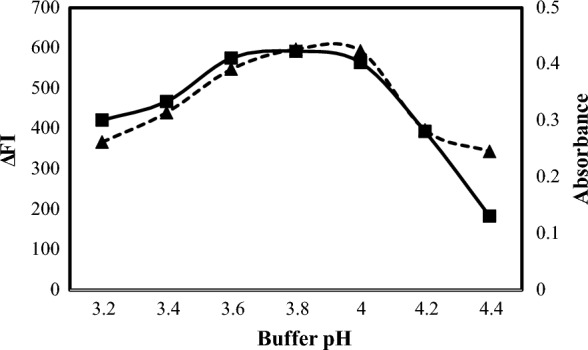
Effect of acetate buffer pH on the reaction of eosin (2.5 × 10^−5^ M) with 1500 ng mL^−1^ ALC for the spectrofluorometric method (-▲-) and eosin (1 × 10^−3^ M) with 6 µg mL^−1^ ALC for the spectrophotometric method (-■-)

**Fig. 5:**
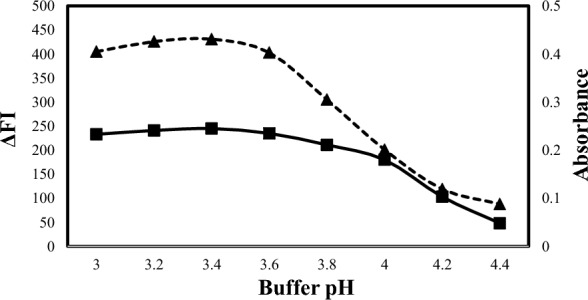
Effect of acetate buffer pH on the reaction of eosin (5 × 10^−4^ M) with 1200 ng mL^−1^ OLO for spectrofluorometric method (-▲-) and eosin (1 × 10^−3^ M) with 6 µ gmL^−1^ OLO for spectrophotometric method (-■-)

An acidic medium should be used to enable the interaction of ALC or OLO with eosin Y. Hence, the pH solution was categorized as a critical variable. The effect of pH on the formation of the ion-pair complex was investigated in the pH range of 3.2–4.6 for ALC (methods A and B) (Fig. 4) and in the pH range of 3.0–4.4 for OLO (methods C and D) (Fig. 5). for ALC, the maximum fluorescence quenching was achieved in pH ranging from 3.6 to 4.0, so pH 3.8 was selected for method A and the maximum absorbance was achieved in pH ranging from 3.6 to 4.0, so pH 3.8 was selected for method B. For OLO, the maximum fluorescence quenching and absorbance were achieved in pH ranging from 3.2 to 3.4, so pH 3.3 was selected for methods C and D.

**Fig. 6:**
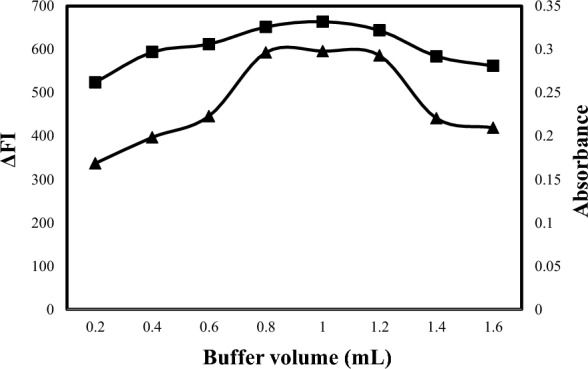
Effect of acetate buffer volume on the reaction of eosin (2.5 × 10^−5^ M) with1500 ng mL^−1^ALC for the spectrofluorometric method (-▲-) and eosin (1 × 10^–3^ M) with 6 µg mL^−1^ ALC for the spectrophotometric method (-■-)

**Fig. 7:**
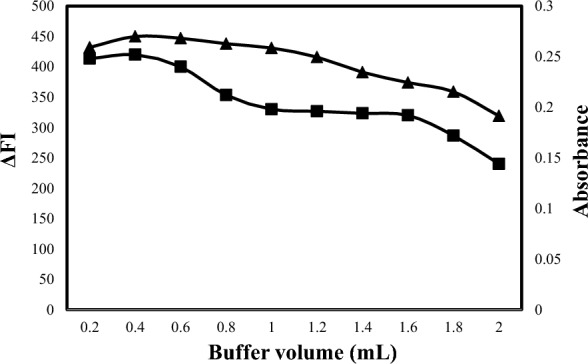
Effect of acetate buffer volume on reaction of eosin (5 × 10^–4^ M) with 1200 ng mL^−1^ OLO for spectrofluorimetric method -▲- and eosin (1 × 10^–3^ M) with 6 µg mL^−1^ OLO for spectrophotometric method -■-

In addition, various buffer volumes ranging from 0.2 to 1.6 mL were examined in methods A and B to determine the most suitable volume (Fig. 6). According to the obtained results, 1.0 ± 0.2 mL of 0.1 M acetate buffer was the appropriate volume for both methods. Also, various buffer volumes ranging from 0.2 to 2.0 mL were examined in methods C and D to determine the most suitable volume (Fig. 7). According to the obtained results, 0.3 ± 0.1 mL of 0.1 M acetate buffer was the appropriate volume for both methods.

#### Effect of the volume of eosin Y

**Fig. 8:**
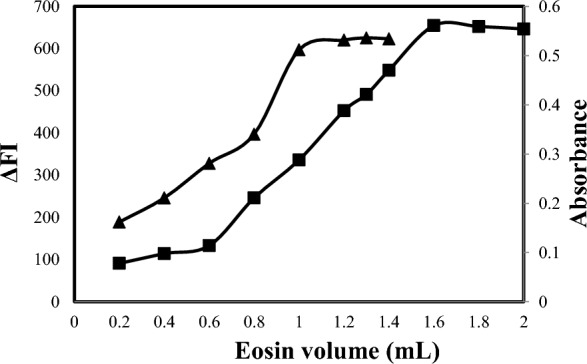
Effect of eosin (2.5 × 10^–5^ M) volume on reaction with ALC 1500 ng mL^−1^ for spectrofluorometric method (-▲-) and eosin (1 × 10^–3^ M) with ALC 6 µg mL^−1^ for spectrophotometric method (-■-)

**Fig. 9:**
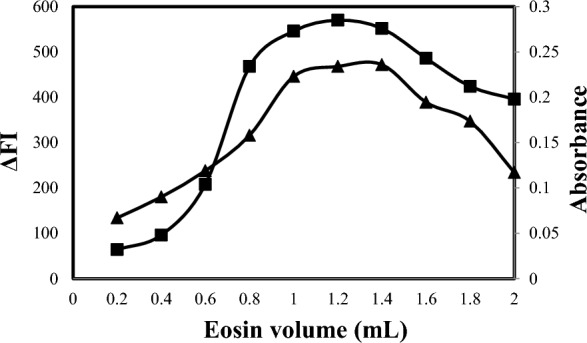
Effect of eosin volume on reaction of eosin (5 × 10^–4^ M) with 1200 ng mL^−1^ OLO for spectrofluorimetric method -▲- and eosin (1 × 10^–3^ M) with 6 µg mL^−1^ OLO for spectrophotometric method -■-

Suitable concentrations of eosin Y that gave the highest results effect were selected for the four methods. Various volumes of the eosin Y reagent were thoroughly tested (Fig. 8) for methods A and B and (Fig. 9) for methods C and D. For method A, the optimum volume of eosin Y was found to be 1.2 mL using solutions with a concentration of 2.5 × 10^–5^ M. In the case of method B, the optimum eosin Y volume was found to be 1.7 mL using solutions with a concentration of 1.0 × 10^–3^ M. For method C, the optimum volume of eosin Y was found to be 1.3 mL using solutions with a concentration of 5 × 10^–4^ M. For method D, the optimum eosin Y volume was found to be 1.2 mL using solutions with a concentration of 1.0 × 10^–3^ M.

#### Effect of diluting solvent

Water, methanol, ethanol, acetone, dimethyl formamide, and acetonitrile were used to dilute the association complex between ALC and eosin Y (Additional file 1: Fig. S1) and OLO and eosin Y (Additional file 1: Fig. S2). Optimum results were obtained when water was used as diluting solvent for all methods. This is a great advantage of the present work since water is environmentally friendly, inexpensive, and readily available.

#### Reaction time between ALC or OLO and eosin Y

The effect of reaction time on the response between the ALC or OLO and eosin Y were investigated. It was observed that the interaction between ALC or OLO and eosin Y was spontaneous for all methods and the reaction was stable for 60 min (Additional file 1: Fig. S3, S4).

### The stoichiometric ratio between ALC or OLO and eosin Y

#### The stoichiometric ratio between ALC and eosin Y

**Fig. 10:**
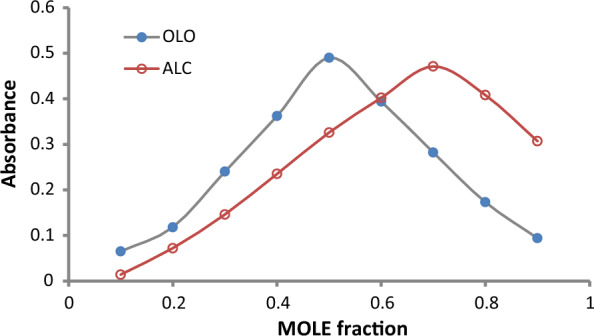
Continuous variation plot for the ion association of ALC or OLO with eosin Y. (the concentration used is 1.3 × 10^–3^ M of each)

**Fig. 11:**
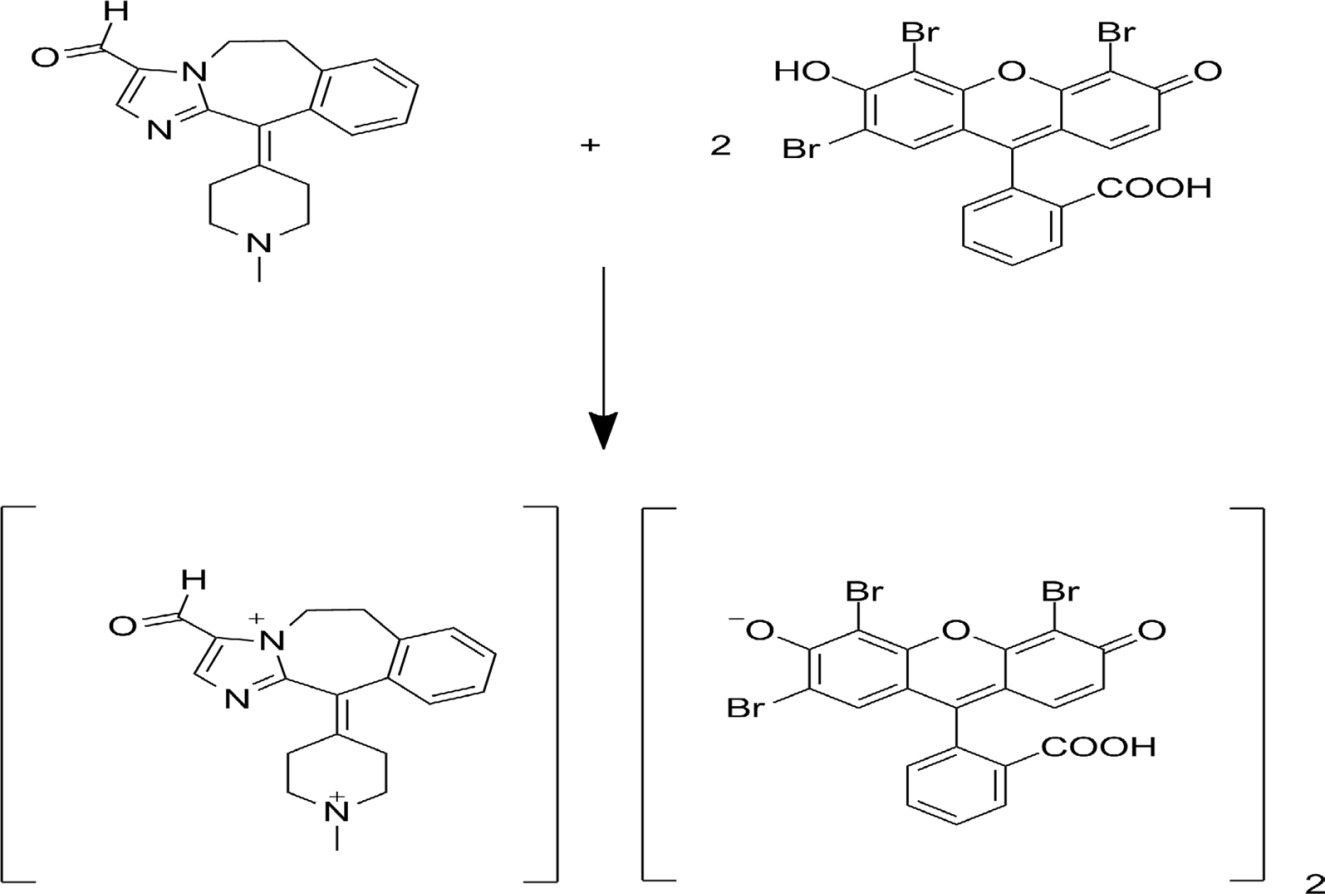
The suggested reaction mechanism for the binary complex formation between ALC and eosin Y

Using (1.3 × 10^–3^ M) master equi-molar solutions, the stoichiometric ratio between the examined ALC and eosin Y was estimated utilizing Job's method (Fig. 10) [30]. The results demonstrated a 2:1 ratio between eosin and ALC indicating that two basic centers (amino groups) in ALC could form the ion pair complex with two molecules of eosin Y. (Fig. 11) depicts a possible reaction mechanism for the association complex formation between ALC and eosin Y. Furthermore, the data of the Job's plot were used to calculate the formation constant (K_f_) of the binary complex using the equation below [31].

$${\text{K}}_{{\text{f}}} = \left( {{\text{A}}/{\text{A}}_{{{\text{ex}}}} } \right)/\left( {{1} - {\text{A}}/{\text{A}}_{{{\text{ex}}}} } \right){\text{C}}^{{\text{n}}} {\text{n}}^{{\text{n}}}$$

where, A is the maximum absorbance, A_ex_ is the extension of the two tangent lines of Job's plot, C is the molar concentration of ALC utilized in Job's approach, and n is the number of the involved moles of ALC in complex formation. The value 3.71 × 10^4^ was the calculated binary complex formation constant (high value indicates high stability). Furthermore, the Gibb's free energy change (∆G_o_) was calculated using the formula: ∆G_o_ = - R T ln K, where; R is the universal gas constant, T is the temperature in Kelvin and K is the constant of the complex formation. The estimated ∆G_o_ was - 2.6 × 10^–4^ J mol^−1^ (negative charge indicates spontaneous reaction).

#### The stoichiometric ratio between OLO and eosin Y

**Fig. 12:**
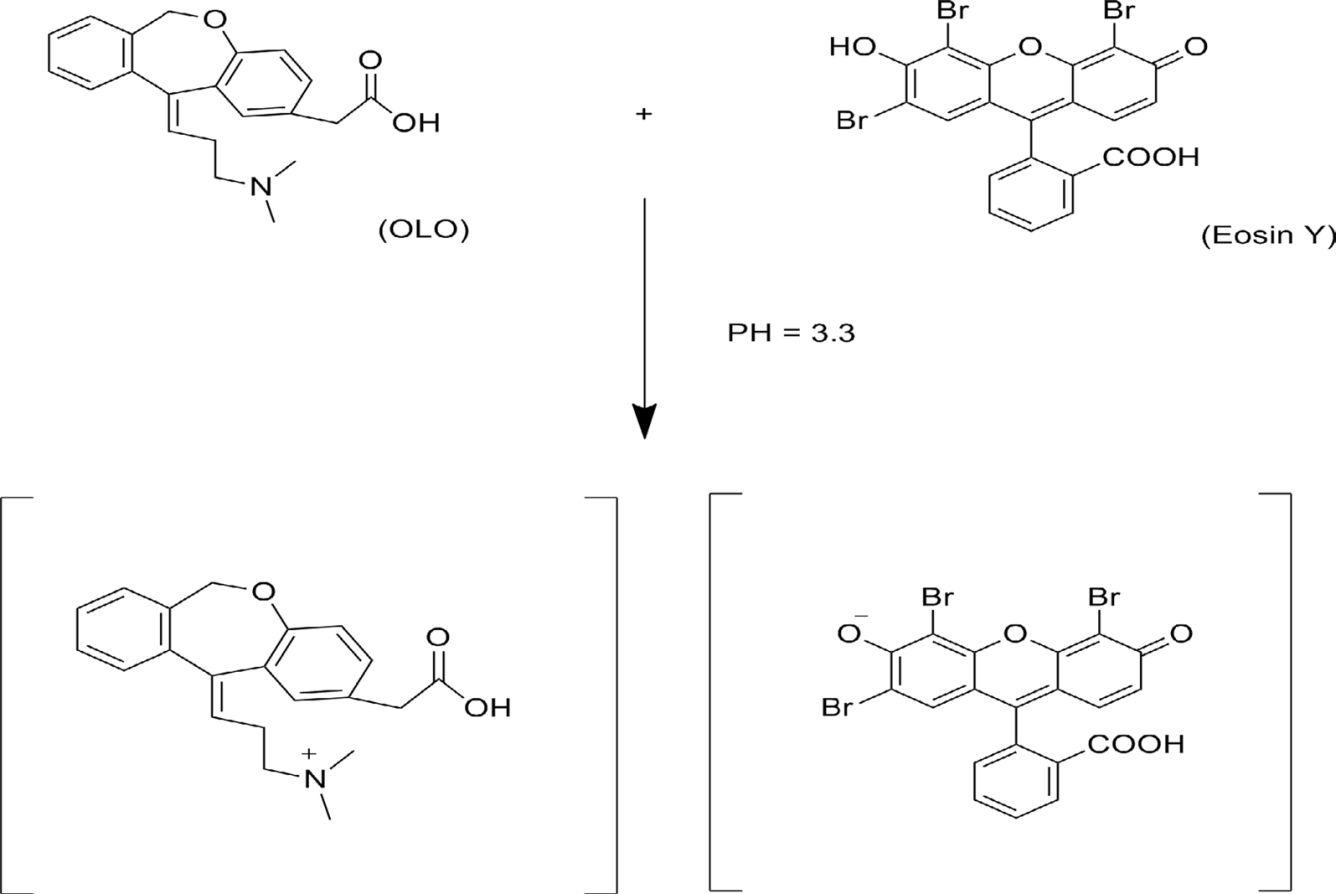
The suggested reaction mechanism for the binary complex formation between OLO and eosin Y

Using (1.0 × 10^−3^ M) master equimolar solutions, the stoichiometric ratio between the examined OLO and Eosin Y was estimated utilizing Job's method (Fig. 10). The results demonstrated a 1:1 ratio between Eosin and OLO indicating that the basic center (amino group) in OLO could form the ion pair complex with one molecule of eosin Y. The possible reaction mechanism for the association complex formation between OLO and eosin Y was illustrated in (Fig. 12). Furthermore, the formation constant (K_f_) was 3.71 × 10^4^ (high value indicates high stability). Furthermore, the Gibb's free energy change (∆Go) was -2.6 × 10^–4^ J mol^−1^ (negative charge indicates spontaneous reaction).

### Methods validation

In accordance with ICH guidelines [32], both methods have been evaluated and validated.

#### Linearity and range

ALC and OLO linearity was achieved using the suggested methods in the concentration range of 150–2000 ng mL^−1^ for method A with a high correlation coefficient (*r*) of 0.9997, 0.8–8.0 µg mL^−1^ for method B with a high correlation coefficient (*r*) of 0.9996, 200–2000 ng mL^−1^ for method C with a high correlation coefficient (*r*) of 0.9996 and of 1.0–10 µg mL^−1^ for method D with a high correlation coefficient (*r*) of 0.9997. The obtained results were subjected to linear regression analysis, and the various analytical parameters were calculated as shown in Table 1.

**Table 1:** The regression and validation parameters for the proposed spectroscopic methods for the determination of ALC and OLO

Parameter	Method A	Method B	Method C	Method D
Linear range (µg mL^−1^)	0.15–2.0	0.8–8.0	0.20–2.0	1.0–10
Slope	0.415	0.091	0.384	0.049
SD of slope (S_b_)	5.10	0.0013	0.005	0.0005
Intercept	0.76	0.0086	0.94	− 0.001
SD of intercept (S_a_)	5.75	0.0059	5.301	0.003
Correlation Coefficient	0.9997	0.9996	0.9996	0.9997
SD of residuals (S_y, x_)	8.20	0.00031	7.56	0.004
LOD	46.67 ng mL^−1^	0.21 µg mL^−1^	45.52 ng mL^−1^	0.21 µg mL^−1^
LOQ	138.40 ng mL^−1^	0.65 µg mL^−1^	137.93 ng mL^−1^	0.63 µg mL^−1^

#### Limit of detection (LOD) and limit of quantification (LOQ)

LOD and LOQ calculation were used to test the sensitivity of both methods. The ICH guidelines equations LOD = 3.3σ/b and LOQ = 10σ/b (b = slope and σ = intercept standard deviation) were used to carry out the calculations. The found detection limits for Method A, B, C and D were 46.67 ng mL^−1^, 0.21 µg mL^−1^, 45.52 ng mL^−1^ and 0.21 µg mL^−1^, respectively, while the quantification limits were 138.40 ng mL^−1^, 0.65 µg mL^−1^, 137.93 ng mL^−1^ and 0.63 µg mL^−1^, respectively, indicating the high sensitivity of the suggested spectroscopic methods.

#### Accuracy and precision

The proposed spectroscopic methods were tested for accuracy using three different concentrations of ALC or OLO, with triplicate measurements for each concentration using the standard addition method. The obtained results show a high degree of agreement between the observed and true values, indicating the proposed spectroscopic methods have high accuracies. Accuracy results were listed in Table 2. all spectroscopic methods were examined for precision (intra- and inter-day precision). The intra- day precision (repeatability) was checked using three different concentrations of ALC or OLO, with triplicate measurements for each concentration within the same day. The inter-day (intermediate) precision was tested over three days using three different concentrations of ALC or OLO, with triplicate measurements for each concentration. Precision results were listed in Table 3. The relative standard deviations for all results were less than 2%, indicating that the proposed methods were very precise.

**Table 2:** Accuracy of the proposed spectroscopic methods for the determination of ALC and OLO using the standard addition method

Amount taken	Amount added	Amount found	% Recovery ± SD^a^
*Method A*			
200 ng mL^−1^	0	198.83 ng mL^−1^	99.42 ± 1.84
200 ng mL^−1^	300 ng mL^−1^	502.24 ng mL^−1^	100.44 ± 1.69
200 ng mL^−1^	800 ng mL^−1^	1014.33 ng mL^−1^	101.43 ± 1.20
200 ng mL^−1^	1300 ng mL^−1^	1502.35 ng mL^−1^	100.16 ± 1.24
*Method B*			
1.0 μg mL^−1^	0	1.001 μg mL^−1^	100.11 ± 1.09
1.0 μg mL^−1^	1.0 μg mL^−1^	2.003 μg mL^−1^	100.02 ± 0.84
1.0 μg mL^−1^	3.0 μg mL^−1^	4.021 μg mL^−1^	100.52 ± 0.72
1.0 μg mL^−1^	5.0 μg mL^−1^	5.994 μg mL^−1^	99.90 ± 1.47
*Method C*			
200 ng mL^−1^	0	200.50 ng mL^−1^	100.25 ± 1.30
200 ng mL^−1^	200 ng mL^−1^	398.24 ng mL^−1^	99.56 ± 0.65
200 ng mL^−1^	600 ng mL^−1^	803.25 ng mL^−1^	100.41 ± 0.68
200 ng mL^−1^	1200 ng mL^−1^	1192.66 ng mL^−1^	99.39 ± 0.33
*Method D*			
2.0 μg mL^−1^	0	2.002 μg mL^−1^	100.11 ± 1.55
2.0 μg mL^−1^	2.0 μg mL^−1^	3.992 μg mL^−1^	99.79 ± 0.78
2.0 μg mL^−1^	4.0 μg mL^−1^	5.995 μg mL^−1^	99.91 ± 1.28
2.0 μg mL^−1^	6.0 μg mL^−1^	8.059 μg mL^−1^	100.73 ± 0.25

^a^Mean of three determination

**Table 3:** Evaluation of the intra-day and inter-day precision of the proposed spectroscopic methods for the determination of ALC and OLO

Method	Conc. level	% Recovery ± RSD^a^	
	μg mL^−1^	Intra-day precision	Inter-day precision
Method A	0.5	100.65 ± 1.84	101.46 ± 1.89
	1.0	101.61 ± 1.43	100.95 ± 1.73
	1.5	100.57 ± 1.25	99.82 ± 1.48
Method B	1.0	100.38 ± 1.63	100.72 ± 1.64
	4.0	101.75 ± 1.19	100.76 ± 1.41
	6.0	100.11 ± 1.28	99.94 ± 1.01
Method C	0.4	98.11 ± 1.35	99.20 ± 1.40
	0.8	100.41 ± 1.66	100.03 ± 1.14
	1.2	100.62 ± 0.78	99.80 ± 1.35
Method D	2.0	101.45 ± 1.00	100.11 ± 1.68
	6.0	99.35 ± 1.42	98.11 ± 1.26
	10.0	99.26 ± 1.14	99.33 ± 1.85

^a^Mean of three determination

#### Robustness

The robustness of the proposed method was carried out to evaluate the influence of small variation in the reaction conditions including; Eosin Y volume, pH, and buffer volume were examined. It was found that none of these variables significantly affect the percentage recovery of ALC or OLO. The results in Table 4 indicate the reliability of the proposed methods during normal use of the methods in the determination of ALC or OLO. So, the methods are considered robust.

**Table 4:** Robustness of the proposed spectroscopic methods for the determination of ALC and OLO

Parameter		% Recovery ± SD^a^			
		Method A^b^	Method B ^b^	Method C^b^	Method D^b^
Buffer pH	− 0.1	100.16 ± 1.25	99.61 ± 0.69	99.75 ± 0.22	98.38 ± 1.13
	+ 0.1	99.68 ± 1.31	99.79 ± 0.57	100.26 ± 0.45	98.65 ± 1.47
Buffer volume	− 0.1 mL	99.03 ± 0.56	99.15 ± 0.27	100.18 ± 0.65	98.85 ± 1.11
	+ 0.1 mL	99.51 ± 0.49	99.52 ± 0.84	99.89 ± 0.25	100.20 ± 0.77
Eosin volume	− 0.1 mL	98.23 ± 0.33	99.43 ± 0.72	99.17 ± 1. 09	98.17 ± 0.91
	+ 0.1ML	100.62 ± 1.07	100.16 ± 1.04	100.04 ± 1.52	98.44 ± 1.57

^a^The values are the mean of three determinations

^b^Drug concentration is 1500 ng mL^−1^ in method A, 4.0 μg mL^−1^ in method B, 1200 ng mL^−1^ in method C and 6.0 μg mL^−1^ in method D

### Pharmaceutical application (ANOVA)

The developed method was applied to detect ALC or OLO in its commercially available eye drops. The obtained percentage of recoveries ± SD (Tables 5 and 6 for ALC or OLO, respectively), were compared with those obtained by the reported method [8, 9]. using the F- and Student's *t*-tests (at 95% confidence level). The results show that the proposed methods can determine the investigated drug (ALC or OLO) in its corresponding pharmaceutical dosage form without any interference from the commonly added excipients. Furthermore, there is no significant difference between the results obtained in this study and the reported method, which indicates that the developed method can be used to analyze ALC or OLO in eye drops with acceptable accuracy and precision.

**Table 5:** Application of the proposed spectroscopic methods for the determination of ALC in Orchinohist® eye drops

Parameters	Reported method [8]	Method A	Method B
% Recovery^a^	100.20	101.05	99.98
Standard deviation, SD	0.55	1.19	0.91
Number of determinations	5	5	5
t-value^a^		1.46	0.81
F-value ^a^		4.72	0.36

^a^Tabulated value at 95% confidence limit; *t* = 2.306 and F = 6.338

**Table 6:** Application of the proposed spectroscopic methods for the determination of OLO in pharmaceutical dosage forms

Dosage form	% Recovery ± SD^a^		
	Reported method [9]	Method C	Method D
Olohistine® 0.1%	100.35 ± 0.67	100.05 ± 0.60 (*t* = 0.74, F = 1.23)	100.12 ± 0.64 (*t* = 0.56, F = 1.10)
Conjyclear forte® 0.2%	100.45 ± 0.63	100.23 ± 0.74 (*t* = 0.83, F = 1.54)	101.25 ± 0.98 (*t* = 1.54, F = 2.44)
Conjyclear forte® 0.2% SDU	100.02 ± 0.61	99.66 ± 0.76 (*t* = 0.50, F = 1.93)	99.48 ± 0.99 (*t* = 1.04, F = 2.61)

^a^Number of determinations = 5

^b^Tabulated value at 95% confidence limit; *t* = 2.306 and F = 6.338

### Evaluation of method of greenness

Analysts wield considerable influence in safeguarding individuals and the environment against detrimental substances and the discharge of waste emanating from sectors such as chemicals and pharmaceuticals. The advancement and enhancement of green chemistry necessitates ongoing efforts. Various tools are employed to assess the environmental impact or "ecological value" of analytical methods in determining their environmental quality [33]. Two assessment tools were employed to evaluate the environmental friendliness of the current procedure:GAPI [28], and AGREE [29].

**Fig. 13:**
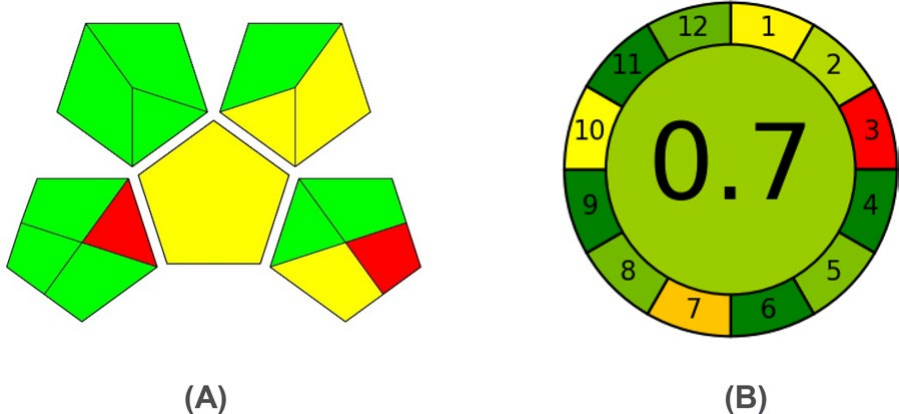
Greenness evaluation of the proposed spectroscopic methods using **A** GAPI and **B** AGREE

GAPI provides qualitative information through pictorial symbols [28]. Using this approach, a thorough evaluation of the environmental effects of the analytical process was undertaken, taking into account particular nuances. Five distinct pentagrams were devised to evaluate specific stages of the analytical procedure that could influence the environment. The assessment employed three different color classifications: green, indicating minimal impact; yellow, suggesting moderate impact; and red, indicating significant impact on the environment. As depicted in Fig. 13A, the GAPI pentagrams indicate that the existing methodologies achieve a satisfactory green rating, with 9 areas highlighted in green, 4 in yellow, and 2 in red.

A newly introduced tool designed for evaluating environmental friendliness is the Analytical Greenness Calculator (AGREE) [29], which provides a user-friendly, adaptable, and thorough method. The software of the calculator is easily accessible, and its results are straightforward to interpret. The outcomes are depicted using pictograms, with the overall score showcased centrally. A score nearing one, such as 0.7, coupled with a green shade at the pictogram's heart, indicates methods' high degree of environmental friendliness. Additionally, the pictogram integrates the twelve tenets of Green Analytical Chemistry (GAC), each depicted by colored segments. A dark green shade denotes maximum environmental friendliness, while red suggests minimal eco-friendliness. Figure 13B presents the AGREE results for the proposed spectroscopic techniques.

## Conclusion

Eosin Y was used as an ion-pairing reagent to form binary complex with ALC and OLO in the current assay. The ion pairing strategy was employed in four spectroscopic methods, two fluorometric and two colorimetric methods. The presented work has the following advantages: it is sensitive, accurate, and precise when it comes to analysis of the aforementioned antihistaminic drug in bulk and commercial pharmaceutical formulation. Furthermore, it is a time-saving approach that eliminates the requirement for sample preparation or extraction. Moreover, it represents the first spectrofluorimetric approach for quantifying ALC and OLO in raw materials and pharmaceutical formulations. The simplicity and sensitivity make these methods excellent candidate for ALC and OLO quality control. The use of distilled water as a green solvent make the present procedure good alternatives for conventional techniques that use harmful organic solvents.

### Supplementary Information

**Additional file 1: Fig. S1.** Effect of diluting solvent on the reaction of eosin (2.5 x 10^-5^ M) with ALC 1500 ng mL^-1^ for the spectrofluorometric method (□) and eosin (1 x 10^-3^ M) with ALC 6 µg mL^-1^ for the spectrophotometric method (■). **Fig. S2. **Effect of diluting solvent on the reaction of eosin (5 x 10^-4^ M) with 1200 ng mL^-1^ OLO for spectrofluorimetric method (□) and eosin (1 x 10^-3^ M) with 6 µg mL^-1^ OLO for spectrophotometric method (■). **Fig. S3.** Effect of reaction time of eosin (2.5 x 10^-5^ M) with ALC 1500 ngmL^-1^ for the spectrofluorometric method (-▲-) and eosin (1 x 10^-3^ M) with ALC (6 µg mL^-1^) for the spectrophotometric method (-■-). **Fig. S4.** Effect of reaction time of eosin (5 x 10^-4^ M) with 1200 ng mL^-1^ OLO for spectrofluorimetric method -▲- and eosin (1 x 10^-3^ M) with 6 µg mL^-1^ OLO for spectrophotometric method -■-

## Data Availability

All data generated or analyzed during this study are included in this published article.
