# Supplementary material for: Determination of antihistaminic drugs alcaftadine and olopatadine hydrochloride via ion-pairing with eosin Y as a spectrofluorimetric and spectrophotometric probe: application to dosage forms
Source: BMC Chem. 2024 Feb 22;18(1):40. doi: 10.1186/s13065-024-01137-y (PMC10885458; doi:10.1186/s13065-024-01137-y)
Supplement: Supplementary file 1 — Additional file 1: Fig. S1. Effect of diluting solvent on the reaction of eosin (2.5 x 10^-5^ M) with ALC 1500 ng mL-1 for the spectrofluorometric method (□) and eosin (1 x 10^-3^ M) with ALC 6 µg mL-1 for the spectrophotometric method (■). Fig. S2. Effect of diluting solvent on the reaction of eosin (5 x 10^-4^ M) with 1200 ng mL-1 OLO for spectrofluorimetric method (□) and eosin (1 x 10^-3^ M) with 6 µg mL-1 OLO for spectrophotometric method (■). Fig. S3. Effect of reaction time of eosin (2.5 x 10^-5^ M) with ALC 1500 ngmL-1 for the spectrofluorometric method (-▲-) and eosin (1 x 10^-3^ M) with ALC (6 µg mL-1) for the spectrophotometric method (-■-). Fig. S4. Effect of reaction time of eosin (5 x 10^-4^ M) with 1200 ng mL-1 OLO for spectrofluorimetric method -▲- and eosin (1 x 10^-3^ M) with 6 µg mL-1 OLO for spectrophotometric method -■- [file 13065_2024_1137_MOESM1_ESM.docx]

**Determination of Antihistaminic Drugs Alcaftadine and Olopatadine Hydrochloride via Ion-Pairing with Eosin Y as a Spectrofluorimetric and Spectrophotometric Probe: Application to Dosage Forms.**

Sayed M. Derayea ^a^, Khalid M. Badr El-din ^a^, Ahmed S. Ahmed ^b*^, Ahmed A. Khorshed ^b,^ *^c^*, Mohamed Oraby ^b^.

*^a^ Department of Pharmaceutical Analytical Chemistry, Faculty of Pharmacy, Minia University, Minia 61519, Egypt.*

*^b^ Department of Pharmaceutical Analytical Chemistry, Faculty of Pharmacy, Sohag University, Sohag 82524, Egypt.*

*^c^ Department of Biomedical Engineering, University of Alberta,* *Edmonton, AB, T6G 1H9, Canada.*

**Additional materials**

**Fig. S1:** Effect of diluting solvent on the reaction of eosin (2.5 x 10^-5^ M) with ALC 1500 ngmL^-1^ for the spectrofluorometric method (□) and eosin (1 x 10^-3^ M) with ALC 6 µgmL^-1^ for the spectrophotometric method (■).

**Fig. S2:** Effect of diluting solvent on the reaction of eosin (5 x 10^-4^ M) with 1200 ng mL^-1^ OLO for spectrofluorimetric method (□) and eosin (1 x 10^-3^ M) with 6 µg mL^-1^ OLO for spectrophotometric method (■).

**Fig. S3:** Effect of reaction time of eosin (2.5 x 10^-5^ M) with ALC 1500 ngmL^-1^ for the spectrofluorometric method (-▲-) and eosin (1 x 10^-3^ M) with ALC (6 µgmL^-1^) for the spectrophotometric method (-■-).

**Fig. S4:** Effect of reaction time of eosin (5 x 10^-4^ M) with 1200 ng mL^-1^ OLO for spectrofluorimetric method -▲- and eosin (1 x 10^-3^ M) with 6 µg mL^-1^ OLO for spectrophotometric method -■-
